# Exploring the Effects of *Gracilaria lemaneiformis* Polysaccharides on the Fecal Microbiota and Fecal Metabolites of Fattening Pigs Based on 16S rDNA and Metabolome Sequencing

**DOI:** 10.3390/ani15020153

**Published:** 2025-01-09

**Authors:** Mingyang Jia, Qiang Ma, Hongjun Wang, Xiangzhou Yan, Lei Wang, Baosong Xing, Qingxia Lu, Jing Wang

**Affiliations:** 1 Key Laboratory of Livestock and Poultry Breeding and Nutrition Regulation in Henan Province, Institute of Animal Husbandry, Henanmn Academy of Agricultural Sciences, Zhengzhou 450002, China; mingyangjia@hnagri.org.cn (M.J.); xiangzhouyan@hnagri.org.cn (X.Y.); xingbaosong@hnagri.org.cn (B.X.); 2 College of Animal Science and Technology, Henan Institute of Science and Technology, Xinxiang453003, China; lei.wang@hist.edu.cn; 3 Animal Disease Prevention and Control Center of Xin’an County, Luoyang 471800, China

**Keywords:** fattening pigs, *gracilaria lemaneiformis* polysaccharides, 16S rDNA sequencing, untargeted metabolomics

## Abstract

Sixty Duroc × (Landrace × Yorkshire) castrated male finishing pigs were randomly divided into negative control (NC) and *gracilaria lemaneiformis* polysaccharides (GLP) groups to study the effects of GLP on the fecal microbiota and fecal metabolites of fattening pigs. The NC group was fed a basic diet, and the GLP group was fed a basic diet supplemented with 0.1% GLP. The diversity of the species and the fecal metabolites were analyzed using 16S rDNA sequencing and metabolome sequencing, respectively. The serum immune and antioxidant indices were analyzed using the ELISA assay. The abundances of hazardous bacteria such as Proteobacteria at the phylum level and *Shigella* at the genus level were extremely significantly decreased (*p* < 0.01) and those of beneficial bacteria such as Firmicutes at the phylum level (*p* < 0.01), *Clostridium* at the genus level (*p* < 0.01), and *Lactobacillus* at the genus level (*p* < 0.05) were significantly increased in the GLP group compared with the NC group. A total of 41 differentially expressed metabolites were identified. The expression of anti-inflammatory and antioxidant active substances, such as methyl cinnamate, protopanaxatriol, and isovanillic acid, was elevated in the GLP group. The ELISA assay showed increased GSH-Px activity (*p* < 0.01), T-AOC (*p* < 0.01), IgG (*p* < 0.01), IgA (*p* < 0.05), and IgM (*p* < 0.05) in the GLP group. These results indicate that dietary GLP supplementation can improve the antioxidant ability, anti-inflammatory ability, and immune level of fattening pigs by regulating fecal flora and metabolites and could be used as a functional feed additive.

## Simple Summary

Pork is one of the most important animal products consumed by humans. With antibiotic bans becoming stricter and feed costs increasing, the search for substitute feed additives that can promote animal growth, improve animal immunity, and increase feed utilization rates has become urgent. *Gracilaria lemaneiformis* polysaccharide (GLP) is an important functional component of *gracilaria lemaneiformis*, which has various biological functions, such as immune-enhancing, antioxidant, intestinal microbiota regulation, lipid metabolism regulation, etc. In order to analyze whether GLP could be used as a feed additive for fattening pigs, we added a supplementation of 0.1% GLP to the diet of fattening pigs, collected their feces and serum after 40 days of feeding, and analyzed the effects of dietary GLP on fecal microbiota and fecal metabolites using 16S rDNA sequencing, metabolomic sequencing, and ELISA methods. This study is expected to provide a reference for developing new functional feeds, improving breeding efficiency and promoting food-saving agriculture.

## 1. Introduction

Pork is a primary animal food consumed by humans. The pig industry is facing severe challenges, as the policy of banning antibiotics is becoming stricter and feed costs are increasing. The search for substitute resistance products that can promote animal growth, improve animal immunity, and increase feed utilization rates as feed additives has become urgent in the breeding industry.

The diversity of the intestinal microbiota confers potential biological functions on the host, and a large number of studies have demonstrated that intestinal microbiota played a key role in processes such as nutrient digestion and absorption, pathogen suppression, and immunomodulation [1]. Alterations in the microbiota structure and species composition of intestinal microbiota will affect intestinal health and nutrient digestion and absorption [2]. It has been shown that dietary glycyrrhiza polysaccharide supplementation altered the diversity and community composition of cecal microbiota in pigs, with increasing relative abundance of Bacteroidota and *Lactobacillus* at phylum and genus levels (*p*  < 0 .05), and improved growth performance and intestinal health of weaned piglets [3]. In the gut, undigested and unabsorbed nutrients are fermented by microorganisms to produce a variety of metabolites with different biological activities. Intestinal microorganisms convert macromolecular carbohydrates, fatty acids, and proteins into a variety of metabolites, such as short-chain fatty acids, amino acids, small peptides, polyamines, and bile acid salts. These metabolites play important roles in nutrient metabolism and immune homeostasis [4]. Lee et al. found that modifying the low crude protein diet with resistant starch supplementation modulated concentrations of ileal propionic acid and colonic butyric acid, improved gut morphology and affected growth performance in weaned pigs [5].

*Gracilaria lemaneiformis*, belonging to the family *Gracilariaceae* (*Rhodophyta*), has a long history as a traditional Chinese medicine and is rich in nutrients, such as dietary fiber, protein, and trace mineral elements [6]. *Gracilaria lemaneiformis* polysaccharide (GLP) is an important functional component of *gracilaria lemaneiformis*, which has various biological functions, such as immunity-enhancing, antioxidant, antiviral, antitumor, intestinal microbiota regulation, lipid metabolism regulation, and other functions [7,8,9]. Jiang et al. used GLP as a feed additive to improve the growth performance of broiler chickens by altering their antioxidant capacity, immune function, and intestinal microbiota composition [10]. Our previous study found that the addition of 0.1% GLP to the diet improved growth performance and meat quality in fattening pigs, such as higher average daily gain, lower feed-to-weight ratio, and significantly higher intramuscular fat content [11], but its effects on the fecal microbiota and fecal metabolites in fattening pigs are still unknown. In this study, 16S rDNA sequencing, metabolome sequencing, and ELISA were used to analyze the effects of dietary supplementation with GLP on the fecal microbiota, fecal metabolites, antioxidant capacity, and immune function of castrated male finishing pigs, which provided a reference for the development of new functional feeds, the improvement of breeding efficiency, and the promotion of food-saving farming.

## 2. Materials and Methods

### 2.1. Ethics Statement

The experimental methods were performed following the Good Experimental Practice guidelines adopted by the Institute of Animal Husbandry. All animal procedures in this study complied with the Institutional Animal Care and Use Committee (IACUC) guidelines, authorized by the Henan Academy of Agricultural Sciences’ Institute of Animal Husbandry (Zhengzhou, China, ID: 2023-8[1]).

### 2.2. Experimental Materials

GLP powder was purchased from Shaanxi Huanuo Biotechnology Co. Ltd. (Xi’an, China), and G. lemaneiformis was harvested from Nan’ao Island, Guangdong, China. The GLP powders were incubated overnight in a methanol/dichloromethane/water solution (4:2:1; *v*/*v*/*v*) with shaking to remove low-molecular-weight compounds. The polysaccharide content isolated from G. lemaneiformis was ≥50%, as determined via the phenol–sulfuric acid method [12].

### 2.3. Experimental Design and Feeding Management

The experiment followed a one-way randomized group design, where 60 Duroc × (Landrace × Yorkshire) castrated male finishing pigs (with body weights of 80 kg) were randomly divided into negative control (NC) and GLP groups, with 3 replicates in each group and 10 pigs in each replicate. The NC group was fed a basic diet, and the GLP group was fed a basic diet supplemented with 0.1% GLP. The basic diet was prepared according to the pig nutrition demand scale of the NRC (2012) [13] and the Chinese Pig Breeding Standard (NY/T65-2004) [14]. The diet was powdered compound feed. The composition and nutrient levels of the basic diet are shown in Table 1. The contents of crude protein, crude fiber, crude ash, calcium, phosphorus, and amino acids were detected using the methods of GB/T 6432-2018 [15], GB/T 6434-2022 [16], GB/T 6438-2007 [17], GB/T 6436-2018 [18], GB/T 6437-2018 [19], and GB/T 18246-2019 [20], respectively.

The feeding experiment was carried out at the Xinmi breeding base of the Institute of Animal Husbandry, the Henan Academy of Agricultural Sciences. The experiment was conducted for a total of 50 days, with a pre-experimental period of 10 days and an experimental period of 40 days. The pig house was a traditional semi-open type with natural ventilation. The test pigs were grouped into pens, watered freely, and fed sufficiently twice a day (at 09:00 and 16:00). To ensure the validity of the data, the use of antibiotics was strictly prohibited throughout the experiment. At the end of the experimental period, three pigs from each group were randomly selected, and fresh fecal samples were collected, rapidly frozen in liquid nitrogen for 12 h, and subsequently transferred to −80 °C for storage until the sequencing analysis was performed. Blood samples were collected via venipuncture of the jugular vein into anticoagulant-filled tubes (EDTA) and immediately centrifuged at 3000× *g* for 10 min at 4 °C to obtain serum. Serum samples were stored at −20 °C until further analysis.

### 2.4. Microbiological Analysis

The OMEGA Soil DNA Kit was used to extract total genomic DNA from the fecal samples (M5635-02) (Omega Bio-Tek, Norcross, GA, USA). NanoDrop NC2000 spectrophotometer (Thermo Fisher Scientific, Waltham, MA, USA) and agarose gel electrophoresis were used to measure the quantity and quality of the extracted DNA. The 16s rDNA sequencing were performed by Wuhan Frasergen Biotechnology Corporation (Wuhan, Hubei, China). Amplicons were pooled and 2250 bp paired-end sequenced on the Illumina MiSeq platform using the MiSeq Reagent Kit v3. QIIME2 (version 2019.4) was used for microbiome bioinformatics. The sequences were then quality filtered, denoised, merged and the chimeras removed. Amplicon sequence variants (ASVs) were extracted using the DADA2 plugin [22]. Based on the SILVA Release 132 database, ASVs were assigned to taxonomic groups using the classify-sklearn naive Bayes taxonomy classifier in the feature-classifier plugin [23,24]. Alpha-diversity indices were calculated using the ASV table in QIIME2. They were visualized as box plots. The weighted UniFrac distances were used to calculate the Beta-diversity and the results were visualized using Principal Coordinate Analysis (PCoA). The Kruskal–Wallis rank–sum test was performed to conduct the linear discriminant analysis effect size (LEfSe), with significance set at *p* < 0.05 and a threshold value of 2.0 for the log-linear discriminant analysis (LDA) score.

### 2.5. Untargeted Metabolomics Profiling

Two LC-MS methods were used to analyze all fecal samples [25]. The analytical conditions were 0.4 mL/min of the flow rate, 4 μL of the injection volume, and 40 °C of the column temperature [26]. Using the same elution gradient as in positive ion mode, another aliquot was analyzed under negative ion conditions. Data were acquired using information-dependent acquisition (IDA) and Analyst TF 1.7.1 software (Sciex, Concord, ON, Canada) [27]. Frasergen Co., Ltd. (Wuhan, China) provided the untargeted metabolomics services. ProteoWizard v.3.0.22 software was used to convert the original LC-MS data file into mzXML format (Palo Alto, CA, USA). Using R v.4.3.3 software (ComplexHeatmap v.2.9.4, Heidelberg, Germany; MetaboAnalystR v.1.0.1, Montreal, Canada), metabolites from all samples were used for hierarchical cluster analysis (HCA) and orthogonal partial least squares discriminant analysis (OPLS-DA) to investigate metabolite accession-specific accumulation [28]. Screening conditions were *p*-values less than 0.05 and fold change values greater than 2.0. The differential metabolites were subjected to KEGG enrichment analysis (http://www.kegg.jp/kegg/pathway.html (accessed on 23 November 2024)) [29]. The GraphPad Prism v.6.01 (GraphPad Software Inc., La Jolla, CA, USA) and R software were used to graph the data [30].

### 2.6. Assessment of Antioxidant Enzymes in Serum

The serum antioxidant capacity was determined using the ELISA kit (Nanjing Jiancheng Biological Institute, Nanjing, China), and the procedure was carried out strictly following the instructions. The assays included superoxide dismutase (SOD), glutathione peroxidase (GSH-Px), catalase (CAT), malondialdehyde (MDA), and the total antioxidant capacity (T-AOC). Each sample was determined three times in duplicate.

### 2.7. Measurement of Serum Immune Markers

The serum immune globulin A (IgA), immune globulin G (IgG), and immune globulin M (IgM) concentrations were tested using an enzyme-linked immunosorbent assay kit (Beijing Solaibao Biotechnology Co., Ltd., Beijing, China). Each sample was determined three times in duplicate.

### 2.8. Statistical Analysis

The statistical significance of the differences was evaluated using SPSS 22.0 software, and the results were reported as the means ± SEMs. Comparisons between the two groups were performed using t-tests. Comparisons among multiple groups were performed using one-way ANOVA. Differences were considered significant when * *p* < 0.05 or ** *p* < 0.01.

## 3. Results

### 3.1. Effects of GLP on Fecal Microbiota in Fattening Pigs

#### 3.1.1. Sequencing Information

**Figure 1:**
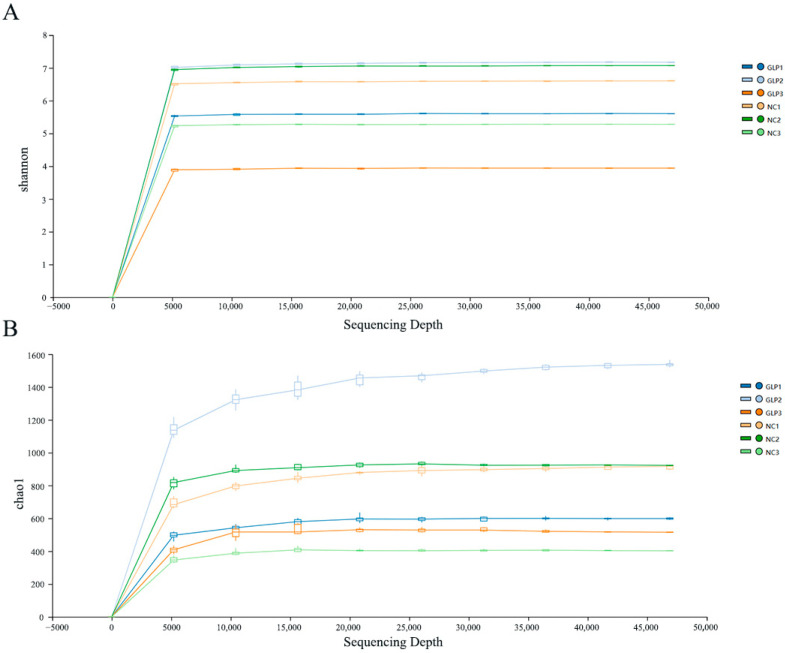
Quality assessment of sequencing data of fecal microbiota from fattening pigs. (**A**) Shannon dilution curve. (**B**) Chao1 dilution curve.

Based on the ASV levels, dilution curve analysis was conducted using the Shannon dilution curve (Figure 1A) and the Chao1 dilution curve (Figure 1B). The results show that the dilution curves tended to flatten and eventually reached a plateau period, indicating that the sequencing depth reached the research standard. The sequencing results fully reflect the diversity contained in these samples. The sequencing depth of the samples covered most of the bacterial species in the intestine. These results were used for the subsequent data analysis.

#### 3.1.2. Alpha-Diversity Analysis

**Figure 2:**
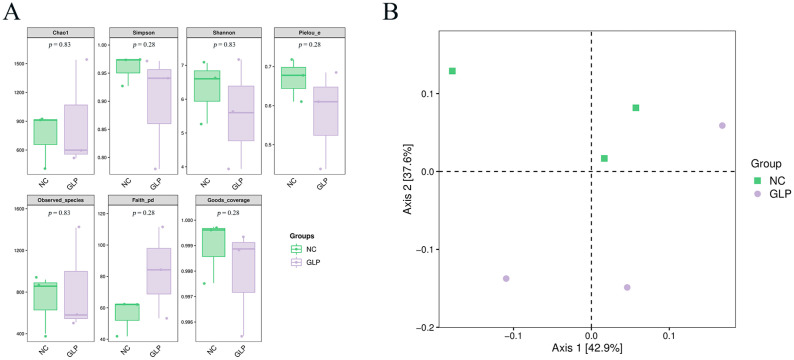
Effect of dietary supplementation with *Gracilaria lemaneiformis* polysaccharides on the diversity of fecal microbiota in fattening pigs. (**A**) Group boxplots of alpha diversity indices. (**B**) Two-dimensional map of sample analyzed using PCoA.

Alpha diversity reflects the species abundance and species diversity of individual samples. In this study, the Chao1 index, Simpson index, Shannon index, observed species index, and Faith’s PD index were used to describe the abundance, diversity, and evolutionary diversity. Pielou’s index and Good’s coverage index were used to characterize the evenness and coverage, respectively. The results of the alpha-diversity analysis suggest that there were no significant changes in the Chao1 index, Shannon index, Simpson index, Pielou’s evenness, observed species index, Faith’s pd index, or Good’s coverage index of the fecal microbiota between the NC and GLP groups (Figure 2A), suggesting that the addition of dietary GLP had no significant effect on the diversity or abundance of the fecal microbiota of the fattening pigs (*p* > 0.05).

#### 3.1.3. Beta-Diversity Analysis

Beta-diversity refers to the diversity between samples and is a measure of the similarity of the microbial composition between individuals. As shown in Figure 2B, the calculation of the distances among the samples only distinguished some of the samples between the two groups. Furthermore, the results of the Adonis test show that the differences in the composition of the fecal microbiota between these two groups were not significant (*p* > 0.05) (Table 2).

#### 3.1.4. Differences in the Structure and Abundance of the Fecal Microbiota

**Figure 3:**
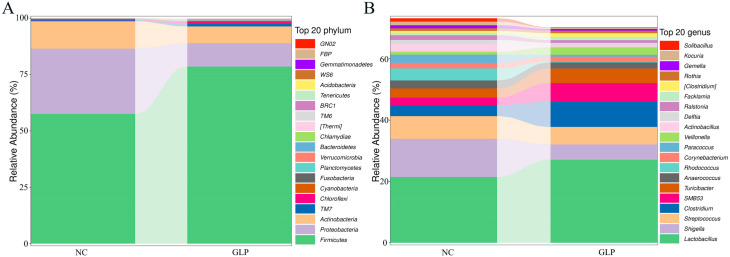
Effect of dietary supplementation with *gracilaria lemaneiformis* polysaccharides on the abundance of fecal microbiota fattening pigs at phylum (**A**) and genus (**B**) level.

In this study, 27 phyla and 419 genera were detected in the NC group, and 28 phyla and 443 genera were detected in the GLP group (Tables S1 and S2). As shown in Figure 3A, at the phylum level, the relative horizontal abundances of Firmicutes, Proteobacteria, and Actinobacteria were more than 1%. The sum of these three bacterial phyla accounted for more than 96% of the total content in both the NC and GLP groups, respectively. The abundance of fecal microbiota in the NC and GLP groups at the genus level are shown in Figure 3B.

As shown in Table 3, the relative abundance of Firmicutes (*p* < 0.01) significantly increased and that of Proteobacteria (*p* < 0.01) significantly decreased compared with the NC group at the phylum level. Meanwhile, the relative abundance of *Shigella* significantly decreased (*p* < 0.01), that of *Clostridium* significantly increased (*p* < 0.01), and that of *Lactobacillus* significantly increased (*p* < 0.05) at the genus level.

#### 3.1.5. Analysis of Shared and Specific Fecal Microbiota

**Figure 4:**
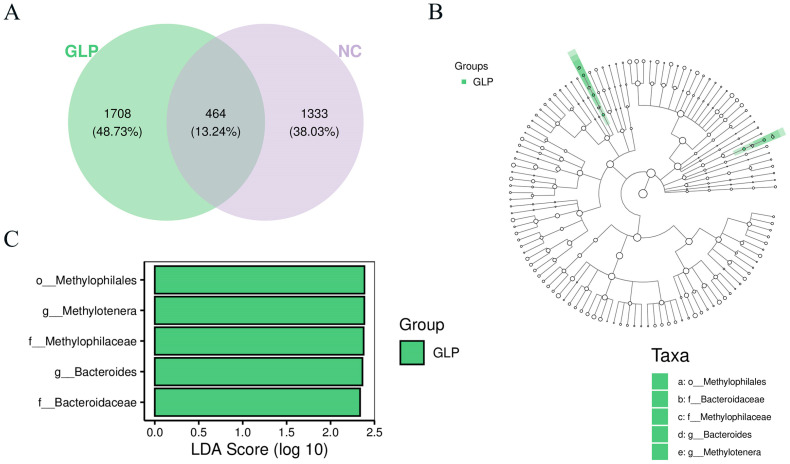
The shared and special fecal microbiota between NC and GLP groups. (**A**) Venn diagram of ASVs (**B**) Branching diagram of LEfSe analytical (**C**) Bar graph of LDA effect values.

As shown in Figure 4A, the NC and GLP groups contained 464 ASVs, accounting for 13.24% of the total ASVs. At the genus level, the abundances of *Lactobacillus*, *Shigella*, *Clostridium*, *Streptococcus*, *SMB53*, and *Turicibacter* were relatively high in both groups. The special fecal microbiota were analyzed using LEfSe (Figure 4B,C). The results show that *o_Methylophilales*, *g_Methylotenera*, *f_Methylophilaceae*, *g_Bacteroides*, and *f_Bacteroidaceae* were the representative bacteria of the GLP group (LDA > 2).

### 3.2. Effects of GLP on Fecal Metabolites in Fattening Pigs

#### 3.2.1. Major Metabolite Profiling

**Figure 5:**
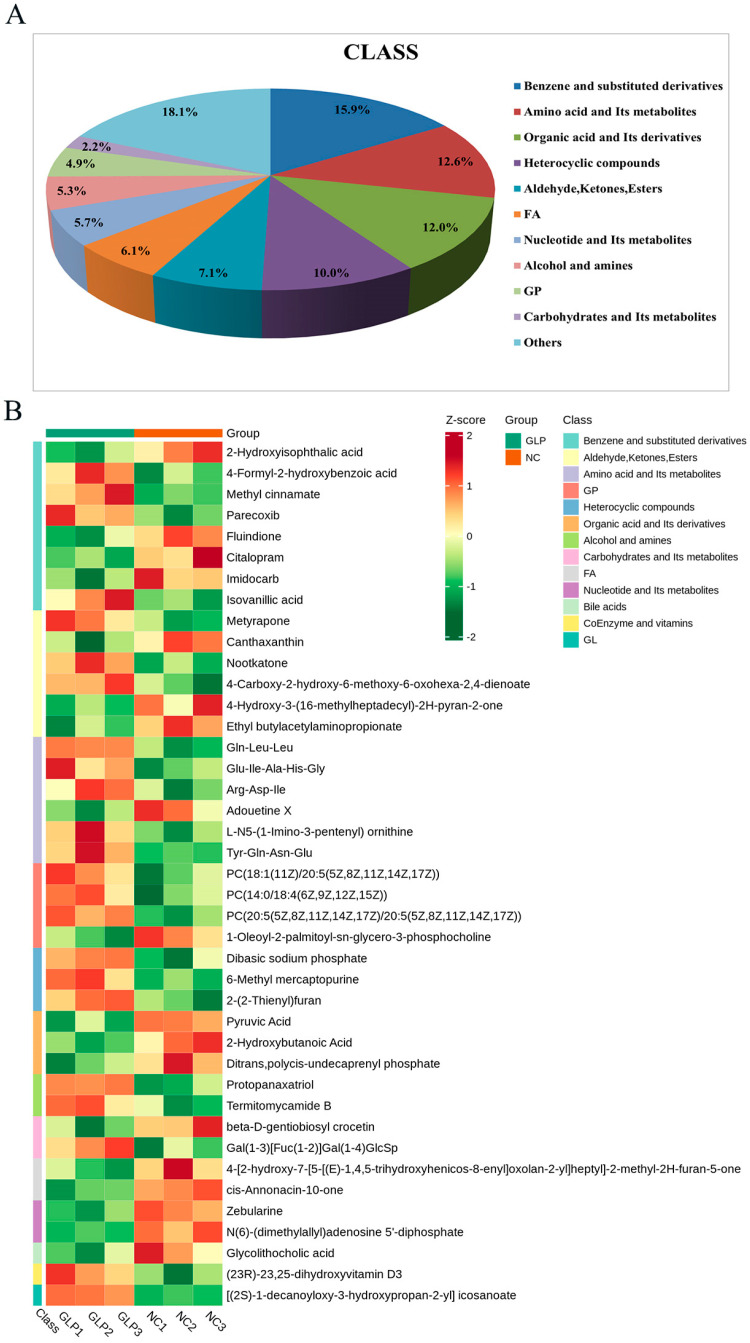
Composition (**A**) and clustering analysis heatmap (**B**) of metabolites in feces from NC and GLP groups. Colors correspond to the distinct values achieved following relative content normalization (red denotes high levels and green denotes low levels).

A thorough examination of the metabolites in the two groups was carried out utilizing untargeted metabolomics with LC-MS to study the effect of GLP on the fecal metabolites of fattening pigs. A comprehensive analysis revealed a total of 2509 metabolites, including 924 metabolites in the negative-ion mode and 1585 metabolites in the positive-ion mode (Table S3). Specifically, these metabolites included 400 benzene and substituted derivatives (15.9%), 316 amino acids and their metabolites (12.6%), 302 organic acids and their derivatives (12.0%), 250 heterocyclic compounds (10.0%), and various additional chemicals (Figure 5A). A quantitative cluster analysis identified distinct metabolomic differences between the NC and GLP groups (Figure 5B).

#### 3.2.2. Differentially Accumulated Metabolite Analysis

**Figure 6:**
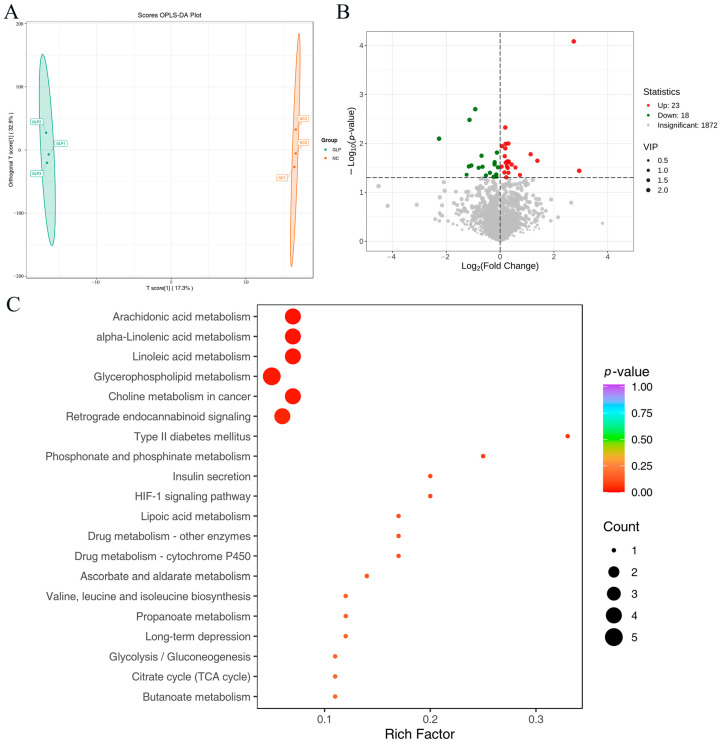
Investigation of differentially accumulated metabolites between the NC and GLP groups. (**A**) Fecal metabolite profiling was performed using OPLS-DA models between NC and GLP groups. (**B**) Volcano graphs showing the differentially accumulated metabolites between NC and GLP groups. The x-axis signifies the Rich Factor associated with each pathway, while the y-axis shows the names of the pathways arranged in order of their *p*-value. (**C**) The KEGG enrichment plots show the metabolic pathways enriched with specific metabolites that are expressed differently between the NC and GLP groups. The color of the data points reflects the size of the *p*-value, where red shades suggest a higher level of enrichment. The magnitude of the data points corresponds to the quantity of metabolites that are differentially expressed and enriched in that particular pathway.

The fecal metabolite samples from the NC and GLP groups were compared pairwise to determine the differentially accumulated metabolites (DAMs). In the OPLS-DA models (Figure 6A), the GLP group was clearly separated from the NC group, indicating significant differences in the properties of the fecal metabolites in the two groups.

All 2509 metabolites were subsequently evaluated for DAMs using the fold change (FC ≥ 2 or ≤ 0.5), the statistical significance of inter-group differences (*p* < 0.05), and the variable importance in the projection (VIP > 1) scores. The screening findings are shown graphically using volcano plots (Figure 6B). For the GLP group, there were 41 DAMs (23 upregulated and 18 downregulated) compared with the NC group (Table S4). These differential metabolites primarily belonged to benzene and substituted derivatives, amino acids and their metabolites, aldehyde, ketones, esters, glycerophospholipids (GPs), organic acids and their derivatives, heterocyclic compounds, carbohydrates and their metabolites, nucleotide and its metabolites, alcohol and amines, fatty acids (FAs), glycerolipids (GLs), bile acids, and coenzyme and vitamins (Table S5). Compared with the NC group, metabolites such as methyl cinnamate, protopanaxatriol, isovanillic acid, and pyruvic acid were significantly upregulated in the GLP group.

#### 3.2.3. Differences in Metabolic Pathways Between NC and GLP Groups

The DAMs’ functions were determined using KEGG pathway analysis (Figure 6C). These differential metabolites were associated with arachidonic acid metabolism, alpha-linolenic acid metabolism, linoleic acid metabolism, glycerophospholipid metabolism, choline metabolism in cancer, and retrograde endocannabinoid signaling.

### 3.3. Effect of GLP on Antioxidant Capacity in Fattening Pigs

The data on the effects of dietary GLP supplementation on the serum levels of antioxidant enzymes are presented in Table 4. Compared with the NC group, the serum MDA concentration decreased, and the SOD, GSH-Px, and CAT activities and total antioxidant capacity increased, with highly significant increases in the GSH-Px activity and total antioxidant capacity (*p* < 0.01) in the GLP group.

### 3.4. Effects of GLP on Serum Immunological Indicators in Fattening Pigs

**Figure 7:**
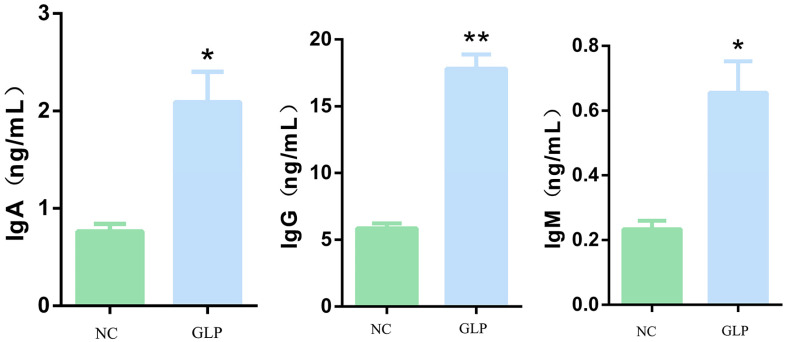
Effect of dietary supplementation with *gracilaria lemaneiformis* polysaccharides on serum immunity index in fattening pigs. * mean significant difference (*p* < 0.05), ** mean extremely significant difference (*p* < 0.01).

Compared with the NC group (Figure 7), GLP supplementation extremely significantly increased the IgG content (*p* < 0.01) and significantly increased the IgA and IgM contents (*p* < 0.05) in the fattening pigs.

## 4. Discussion

The interaction between the host’s intestinal microbiota and immune system plays an important role in the development of disease, the maintenance of intestinal homeostasis, and the promotion of metabolism and development [31,32]. The world has abundant seaweed resources. Polysaccharide, one of seaweed’s main active ingredients, can inhibit the growth and reproduction of harmful bacteria in the intestinal tract and improve intestinal homeostasis, so it is regarded as a natural intestinal microecological regulator [33]. The fecal microbiota can reflect the intestinal microbial community and microecological balance of animals. We analyze the effects of dietary GLP supplementation (0.1%) on fattening pigs via multiple perspectives to provide basic data for developing new feed additives for fattening pigs. In this research, GLP had no significant effect on either microbial alpha-diversity or Beta-diversity (*p* > 0.05), suggesting that it has no negative impact on intestinal health. Therefore, GLP can be used as a routine feed additive for finishing pigs and has wide application prospects.

Previous research has shown that the main phyla of the intestinal microbiota in fattening pigs are Firmicutes, Proteobacteria, and Actinobacteriota [34], which were also the most abundant microbiota communities in this study. Proteobacteria is associated with intestinal inflammatory diseases and is a hallmark species of intestinal microbiota disorders [35]. Bacteria from Firmicutes are predominant in the gut; they belong to the chemotrophic bacteria and can help animals obtain more energy from food [36]. It was found that the higher the abundance of Firmicutes, the higher the feed conversion efficiency of pigs [37]. In this study, the relative abundance of Firmicutes was significantly upregulated (*p* < 0.01) and that of Proteobacteria was significantly downregulated (*p* < 0.01) compared with the NC group. In conjunction with our previous study [11], dietary GLP supplementation resulted in a slight decrease in the feed-to-weight ratio of fattening pigs, indicating an increase in feed conversion efficiency, which aligns with the positive correlation between Firmicutes and the feed conversion ratio. However, this effect must be investigated at higher dosages or longer feeding cycles. The changes in the relative abundance of each dominant bacterial phylum indicated that dietary GLP supplementation resulted in improved digestive ability and gradual intestinal development in fattening pigs.

At the genus level, *Lactobacillus* was the common dominant genus in both groups, and the abundance of *Lactobacillus* in the GLP group was significantly higher than that in the NC group (*p* < 0.05). Studies have shown that *Lactobacillus* reduces intestinal pH, inhibits the growth of pathogenic microorganisms, improves gastrointestinal function, and enhances immunity by utilizing a variety of carbohydrates to produce lactic acid [38]. Compared with the NC group, *Shigella* was highly significantly decreased (*p* < 0.01) and *Clostridium* was highly significantly increased (*p* < 0.01) in the GLP group. *Shigella* is a pathogen, and diarrhea is mainly caused by an increased number of *Shigella* and other pathogenic bacteria [39]. *Clostridium* has protease activity that degrades proteins into peptides, which are further degraded by endopeptidases into oligopeptides and amino acids, which serve as substrates to promote fermentation by intestinal microbes [40]. These results show that GLP increases beneficial bacteria and decreases harmful bacteria.

*Gracilaria lemaneiformis* polysaccharides have a wide range of antioxidant effects with significant health-promoting properties. It alleviated enteritis by modulating gut microbes and the gut barrier in human and mouse studies [41], but its effect on porcine fecal microbiota is unknown. This study’s metabolomic results suggest that GLP addition promoted the elevated expression of anti-inflammatory activities. Methyl cinnamate is the main component of galangal essential oil, which has anti-inflammatory activity [42]. Studies have shown that methyl cinnamate ameliorates gut microbial ecological dysbiosis by inhibiting MAPK signaling, thereby increasing the content of Firmicutes and decreasing the contents of Bacteroidetes and Proteobacteria at the phylum level. At the genus level, it increases the proportion of *Lactobacillus* and decreases the proportion of *Bacteroides* [43]. Protopanaxatriol saponins, the bioactive components of ginseng, increase the concentration of short-chain fatty acids, receptor proteins, and tight-junction proteins, decrease pro-inflammatory cytokines, and enhance intestinal barrier integrity [44]. In a study on mice with a high-fat diet/STZ-induced diabetes, protopanaxatriol reduced the Firmicutes/Phytobacteria ratio and significantly improved intestinal microbiota disruption [45]. Isovanillic acid is found in various plants and possesses anti-inflammatory, antioxidant, and hypolipidemic activities. Isovanillic acid was shown to protect mice against Staphylococcus aureus by targeting vWbp and Coa [46]. Pyruvic acid, a glycolytic metabolite, is a key node in several metabolic pathways, and its dysregulated metabolism can lead to insulin resistance and inflammatory diseases. Moreover, it is significantly correlated with macrophage function and immune responses, and it has been shown that pyruvate can inhibit inflammation and obesity by targeting cPLA2 [47]. In this study, GLP supplementation significantly increased intestinal Firmicutes at the phylum level, significantly downregulated Proteobacteria at the phylum level, and significantly increased *Lactobacillus* at the genus level in fattening pigs. This may be attributed to the elevated levels of methyl cinnamate, protopanaxatriol, isovanillic acid, pyruvic acid, and so on.

In the KEGG enrichment analysis, multiple inflammation-related pathways were significantly upregulated in the GLP group, including arachidonic acid, alpha-linolenic acid metabolism, glycerophospholipid, choline, and other metabolic pathways. Arachidonic acid metabolites play an important role in the development and progression of inflammatory diseases. Multi-target drugs based on the arachidonic acid metabolic pathway have become an important direction in anti-inflammatory drug research. Alpha-linolenic acid has anti-inflammatory and anti-allergic physiological functions. Furthermore, alpha-linolenic acid can reduce inflammation by regulating Th1/Th2 imbalance via the JAK/T-bet/STAT1 and JAK/GATA3/STAT6 pathways [48]. Glycerophospholipid metabolism is a key metabolic pathway in systemic immunity and low-grade inflammatory states [49]. Choline supplementation in weaned piglets alters the intestinal microbiota and affects intestinal inflammation [50]. As a very strong antioxidant, alpha-lipoic acid regulates immunoglobulin and cytokine levels and improves humoral immunity in fattening pigs. In addition, valine (Val), leucine (Leu), and isoleucine (Ile) may be involved in immunoregulation by affecting immunoglobulin activity. In this study, IgA (*p* < 0.05), IgG (*p* < 0.01), and IgM (*p* < 0.05) were significantly upregulated in fattening pigs. We hypothesized that GLP modulates the immune level of fattening pigs via the upregulation of these inflammation-related pathways. This agrees with Md Akibul Hasan Bakky’s findings that supplementing rabbitfish feed with lobster sulfate polysaccharides resulted in a significant reduction in the cumulative mortality rate after a Vibrio parahaemolyticus attack compared with the NC group, suggesting that lobster sulfate polysaccharides have a modulatory effect on immunity [51]. GLP may also have an immunomodulatory function in the Th1 immune response by inhibiting T-cell activation [52].

Meanwhile, arachidonic acid, alpha-linolenic acid, choline, lipoic acid, leucine, isoleucine, etc., are also key substances regulating energy metabolism. Arachidonic acid metabolites play an important role in regulating lipid homeostasis [53]. Choline can regulate lipid metabolism in weaned piglets, which, in turn, affects growth performance [50]. Additionally, lipoic acid improves the carcass traits and meat quality of fattening pigs [54]. Leucine improves ration nitrogen utilization efficiency, promotes protein deposition, and significantly improves carcass quality. A lack of isoleucine in the ration can lead to decreased feed intake and decreased production performance in pigs [55]. In addition, several pathways involved in the regulation of energy metabolism were enriched, such as type II diabetes mellitus, insulin secretion, glycolysis/gluconeogenesis, and the TCA cycle. These results suggest that GLP supplementation significantly affects energy metabolism in fattening pigs, which is consistent with previous studies indicating that dietary supplementation with GLP improved broiler growth performance by altering antioxidant capacity, immune function, and intestinal microbial composition [10].

The antioxidant system can prevent animals from being harmed by free radicals, enhance the immune response, and improve disease resistance and growth performance. T-AOC is a comprehensive indicator of the body’s overall antioxidant capacity, which is an important reflection of the body’s defense system [56]. SOD and GSH-Px are important antioxidant enzymes that regulate the generation and scavenging of free radicals in vivo and can prevent cellular damage by lipid peroxides [57,58]. CAT reduces hydrogen peroxide to water, thus protecting cells from the toxic effects of peroxides [59]. Reactive oxygen species (ROS) can oxidize polyunsaturated fatty acids (PUFAs) in the phospholipid zone of biofilms, and an excess of ROS or a decrease in PUFAs can lead to lipid peroxidation, resulting in the destruction of the biofilm structure [60,61]. In this study, we found that GLP supplementation increased serum SOD, GSH-Px, CAT activities, and the total antioxidant capacity, and decreased the MDA concentration, suggesting that GLP helps increase the activity of antioxidant enzymes. This result was not unexpected, as we found that the differential metabolites were enriched in multiple antioxidant metabolism-related pathways in the above metabolome sequencing. Furthermore, our findings agree with those of broilers wherein GLP can increase the activities of SOD, CAT, and GSH-Px, and decrease MDA levels in the serum and livers of broilers [10], indicating that GLP can also serve as a natural antioxidant for finishing pigs.

## 5. Conclusions

Dietary GLP supplementation helps increase the relative abundance of beneficial bacteria, such as Firmicutes at the phylum level, *Clostridium* at the genus level, and *Lactobacillus*, and decrease the relative abundance of harmful bacteria, such as Proteobacteria at the phylum level and *Shigella* at the genus level in fattening pig manure. It also promotes the elevated expression of anti-inflammatory and antioxidant active substances, such as methyl cinnamate, protopanaxatriol, and isovanillic acid. From this combined with serologic results, it can be inferred that supplementation with 0.1% GLP can improve the antioxidant ability, anti-inflammatory ability, and immune level of fattening pigs by regulating their fecal flora and metabolites, making it an excellent functional feed additive.

## Figures and Tables

**Table 1:** Composition and nutrient levels of the basic diet (air-dried; %).

Ingredients	Content
Corn	58.00
Soybean meal	14.00
Wheat bran	9.00
Grass meal	15.00
Premix ^a^	4.00
Total	100.00
Nutrient levels ^b^	
Digestive energy/(MJ/kg)	11.33
Crude protein	13.13
Crude fiber	9.10
Crude ash	8.30
Calcium	0.60
Phosphorus	0.50
Lysine	0.90

^a^ The premix provided the following per kg of feed: 10,800 IU of VA, 10 mg of VB_1_, 3000 IU of VD_3_, 80 mg of VE, 3000 IU of VK_3_, 20 mg of VB_12_, 200 mg of biotin, 15 mg of D-pantothenic acid, 10 mg of nicotinic acid, 90 mg of Fe (as ferrous sulfate), 25 mg of Cu (as copper sulfate), 100 mg of Zn (as oxide zinc), and 15 mg of Mn (as manganese sulfate). ^b^ The digestive energy was determined as a calculated value, while the others were measured values [21].

**Table 2:** Effect of dietary supplementation with *gracilaria lemaneiformis* polysaccharides on the composition of fecal microbiota in fattening pigs.

Type	Df	Sums of Squares	*R* ^2^	*F*	*p*-Value
Group	1	0.046453	0.256689	1.381328	0.400000
Residual	4	0.134517	0.743311	—	—
Total	5	0.180970	1.000000	—	—

**Table 3:** Effect of dietary supplementation with *gracilaria lemaneiformis* polysaccharides on the abundance of dominant fecal microbiota fattening pigs (%).

Items	NC	GLP	*p*-Value
Firmicutes	57.56 ± 5.13	78.57 ± 2.72	0.00
Proteobacteria	28.98 ± 2.32	10.34 ± 0.35	0.00
Actinobacteriota	12.09 ± 1.08	7.43 ± 1.81	0.09
*Lactobacillus*	21.47 ± 0.20	27.09 ± 0.33	0.02
*Shigella*	12.46 ± 0.65	4.67 ± 0.75	0.00
*Streptococcus*	7.36 ± 1.39	5.36 ± 1.01	0.31
*Clostridium*	3.61 ± 0.47	8.24 ± 0.49	0.00
*SMB53*	2.47 ± 0.54	6.10 ± 2.09	0.05
*Turicibacter*	2.97 ± 0.62	4.72 ± 1.91	0.43

**Table 4:** Effect of dietary supplementation with *gracilaria lemaneiformis* polysaccharides on antioxidant activities in fattening pigs.

Items	NC	GLP	*p*-Value
SOD, U/mL	64.91 ± 1.10	69.48 ± 3.30	0.24
GSH-Px, U/mL	599.80 ± 9.18	680.81 ± 17.37	0.00
CAT, U/mL	118.63 ± 8.83	131.80 ± 5.92	0.24
MDA, nmol/mL	0.812 ± 0.15	0.74 ± 0.09	0.70
T-AOC, U/mL	6.92 ± 0.30	10.08 ± 0.68	0.00

## Data Availability

The data presented in the study are deposited in the figshare database (https://doi.org/10.6084/m9.figshare.27643830, https://doi.org/10.6084/m9.figshare.27916782).

## Supplementary Materials

The following supporting information can be downloaded at: https://www.mdpi.com/article/10.3390/ani15020153/s1, Table S1: The abundance of fecal microbiota in fattening pigs at the phylum level; Table S2: The abundance of fecal microbiota in fattening pigs at the genus level; Table S3: Information of metabolites detected in different ion modes; Table S4: Type of differentially accumulated metabolites; Table S5: Number of differentially accumulated metabolites class.
